# Cost-utility of tiotropium in patients with severe asthma

**DOI:** 10.1186/s12962-023-00508-x

**Published:** 2024-01-18

**Authors:** Jefferson Antonio Buendía, Diana Guerrero Patiño

**Affiliations:** 1 Research Group in Pharmacology and Toxicology “INFARTO”, Department of Pharmacology and Toxicology, University of Antioquia, Medellín, Colombia; 2 Hospital Infantil Concejo de Medellín, Medellín, Colombia; 3 Facultad de Medicina, Universidad de Antioquia, Carrera 51D #62-29, Medellín, Colombia

**Keywords:** Tiotropium, Uncontrolled asthma, Cost-effectiveness analysis, Decision analysis, Markov model

## Abstract

**Summary:**

Add-on therapy with tiotropium was cost-effective when added to usual care in patients who remain uncontrolled despite treatment with medium or high-dose ICS/LABA in a middle-income country.

**Background:**

A significant proportion of asthma patients remain uncontrolled despite inhaled corticosteroids and long-acting beta-agonists. Some add-on therapies, such as tiotropium bromide, have been recommended for this subgroup of patients. This study aimed to assess the cost-effectiveness of tiotropium as an add-on therapy to inhaled corticosteroids and long-acting b2 agonists for patients with severe asthma.

**Methods:**

A probabilistic Markov model was created to estimate the cost and quality-adjusted life-years (QALYs) of patients with severe asthma in Colombia. Total costs and QALYs of two interventions include standard therapy with inhaled corticosteroids and long-acting bronchodilators versus add-on therapy with tiotropium. Multiple sensitivity analyses were conducted. Cost-effectiveness was evaluated at a willingness-to-pay value of $5180.

**Results:**

The expected incremental cost per QALY (ICER) is estimated at US$–2637.59. There is a probability of 0.77 that tiotropium + ICS + LABA is more cost-effective than ICS + LABA at a threshold of US$5180 per QALY. The strategy with the highest expected net benefit is Tiotropium, with an expected net benefit of US$800. Our base-case results were robust to parameter variations in the deterministic sensitivity analyses.

**Conclusion:**

Add-on therapy with tiotropium was cost-effective when added to usual care in patients who remain uncontrolled despite treatment with medium or high-dose inhaled corticosteroids and long-acting bronchodilators. Our study provides evidence that should be used by decision-makers to improve clinical practice guidelines and should be replicated to validate their results in other middle-income countries.

## Background

Asthma is a disease that affects more than 300 million people worldwide [1]. Trends suggest increasing asthma prevalence globally, with an anticipated 100 million new cases in the next decade, principally in developing countries [2]. For example, in Colombia, a nationwide Colombian study estimated a prevalence of 6.3%, which is above that of many Latin American countries [3]. Among chronic diseases, asthma is one of the main contributors to increased healthcare expenditures. In the United States, during the next 20 years, the direct costs of asthma in adolescents and adults will likely be over $1.5 trillion [4].

At least 24% of the patients with asthma are classified as severe asthma requiring high doses of inhaled corticosteroids (ICS)-long-acting beta2-agonist (LABA) or ICS-LABA or oral corticosteroids (OCS) [5]. Indeed, despite these drugs, almost 70% of these patients do not achieve total control of symptoms [5]. The direct cost of severe asthma per patient is three times higher than that of mild asthma, which would be higher if we included indirect costs [6, 7]. In this sense, severe asthma is a serious problem for health systems. In the US, Yaghoubi and colleagues calculate that 175 million people will have uncontrolled asthma. If all those people with uncontrolled asthma in the United States can achieve and maintain asthma control, the savings would be about $300 billion in direct costs and $660 billion in indirect costs, recovering 15,462 quality-adjusted life-years [4].

In the last 20 years, new pharmacological alternatives have been developed for patients with severe asthma, including the addition of long-acting muscarinic antagonists (LAMA), such as tiotropium, to the current treatment with ICS-LABA [7]. LAMA has improved lung function and quality of life, increasing the time to severe exacerbation requiring OCS [8–10]. Recent clinical guidelines recommend adding tiotropium to treatment with ICS-LABA in severe asthma [7]. However, this recommendation raises concerns that the extra benefit offered by this drug outweighs the additional cost compared to therapy with only ICS-LABA. This question is even more relevant in developing countries with an increasing prevalence of asthma and constrained healthcare. In Colombia in a retrospective study on 10,706 people diagnosed with asthma between 2017 and 2019, found salbutamol was the most commonly used inhalator by asthma patients in Colombia. This finding constitutes a possible deviation from overuse with a very high frequency of SABAs and not LABAs as the international recommendations for asthma management suggest regarding bronchodilators. 12.5% of patients (n = 495) received triple therapy (ICS/LABA + LAMA [long-acting antimuscarinic]), particularly fluticasone/salmeterol + tiotropium [11]. An economic evaluation of these new drugs could provide evidence to optimize the efficiency of using financial resources in these countries. This study aimed to assess the health and economic consequences of the three strategies of continuing standard therapy and add-on therapy with tiotropium for treating severe asthma in Colombia.

## Materials and methods

**Fig. 1:**
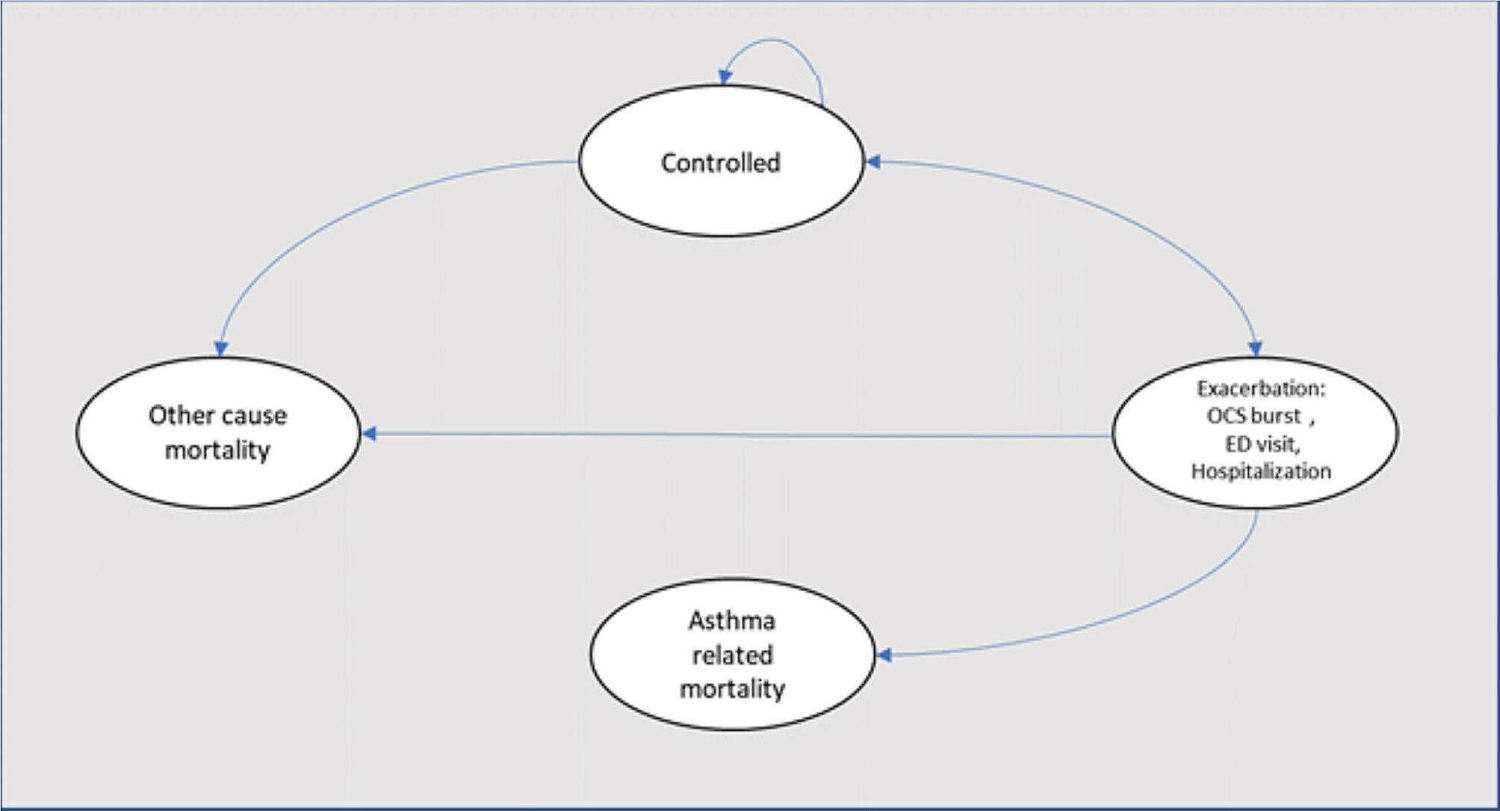
Markov model

**Fig. 2:**
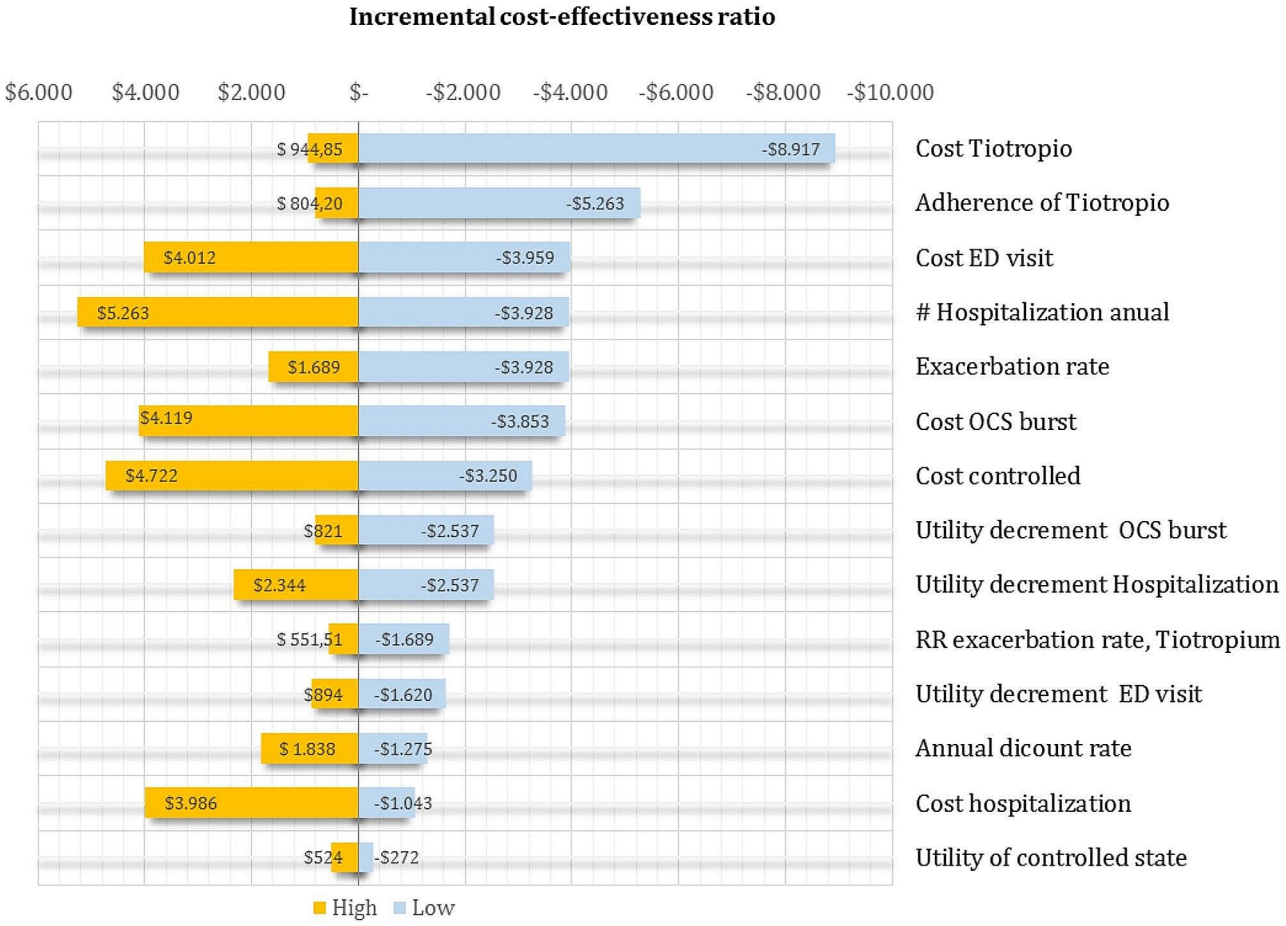
Tornado diagram

We conducted a Markov model to estimate the cost and quality-adjusted life-years (QALYs) of patients > 18 years with severe asthma treated with tiotropium combined with medium-dose ICS/LAB in Colombia. The choice of time horizon was a lifetime. Two interventions were modeled: ICS + LABA and add-on therapy with tiotropium. In this mathematical model, the patients could transition between four mutually exclusive health states (symptom-free state or asthma controlled on tiotropium and SOC and death). During each cycle, patients in non-death health states could transit to any of three levels of asthma exacerbations: OCS burst (was defined as asthma symptoms at least one week and needed to use of oral corticosteroids, prescribed by a physician to achieve the control of symptoms), emergency department (patients who experience an acute asthma attack and are treated in the emergency department with systemic corticosteroids) and hospitalization (Patient whom the physician decides to hospitalize due to failure of initial emergency room management). Asthma-related mortality following an exacerbation or all-cause mortality could also occur (Fig. 1). We made this analysis from a societal perspective (including direct and indirect costs), using a cycle length of 2 weeks. Half-cycle correction and an annual discounting rate of 5% were applied to both costs and QALYs. Cost-effectiveness was evaluated at a willingness-to-pay (WTP) value of US$5180 [12] (Fig. 2).

### Parameters of the Markov model

Multiple parameters were derived from published research and local data presented in Table 1. Relative risk (RR) data on exacerbation rate were extracted from a recent cost-effectiveness study of tiotropium in patients with severe asthma [13]. In this study, the relative risk was 0.72 (CI 95% 0.62–0.83), as from a comparison of the incidence of exacerbation between patients with severe asthma treated with tiotropium combined with medium-high doses of ICS/LABA (n = 274) versus management with only medium-high amounts of ICS/LABA (n = 822). Asthma exacerbations in this study were defined as the use of systemic corticosteroid burst (or SCS, outpatient visits with at least three days of high-dose oral corticosteroids (OCS) or a single SCS injection), emergency department (ED) or hospitalization.

**Table 1:** Model parameters TA

Variable	Inputs used in the sensitivity analysis			References
	Base case	Upper range	Lower range	
Cost tiotropium (per 4 week cycle) (U$)	71	89	53	
Annual cost of health state asthma controlled (U$)	38	47	28	[19]
Annual cost of health state OCS burst (per episode) (U$)	38	47	28	
Annual cost of health state ED visit (per episode) (U$)	2648	3310	1986	
Annual cost of health state hospitalization (per episode) (U$)	230	288	173	
**Utilities (annual)**				
Utility of controlled state	0.740	0.93	0.56	[13]
**Utility decrement**				
Exacerbations requiring OCS burst	0.1	0.13	0.08	[14]
Exacerbations requiring ED visit	0.15	0.19	0.11	
Exacerbations requiring hospitalization	0.2	0.25	0.15	
**Tiotropium effect**				
Relative risk on exacerbation rate	0.72	0.83	0.62	[12]
**Exacerbations**				
Exacerbation anua rate	1.1	4	1	
# Hospitalization annual	2.6	5	1	
**Adherence of tiotropium**	61%	76%	57%	[18]
**Annual discount rate**	5.00%	6%	0%	

*OCS* oral corticosteroids, *ED* emergency

The transition probabilities for moving between different health states for the standard therapy and add-on therapy were derived from clinical trials and economic evaluations of tiotropium [14]. Data of utilities of each Markov state were extracted from a systematic review of utilities in asthma [15, 16] (Table 1). This systematic review identifies a total of 20 studies on asthma that report utilities in different severity states of asthma. These four studies (n = 330 patients) show a median utility of 0.74 ± 0.029 for severe asthma, all estimated using time trade-off or standard gamble or Ashma symptom utility index in the US and UK population. Since all these data (RR, transition probabilities, and utilities) do not come from the Colombian people, they were subjected to probabilistic sensitivity analysis as detailed below and as recommended by the Consolidated Health Economic Evaluation Reporting Standards (CHEERS) Statement [17]. In this sensitivity analysis, to build the range of RR to be used, we use the CI 95% of RR published by clinical trials and in real-life studies [8, 13]. Regarding utilities and transition probabilities, the upper and lower range was estimated by adding or subtracting 25% of the value from the central value defined for the base case. The risk of mortality from other causes was estimated using age- and gender-specific Colombian life tables for all-cause mortality over five years (2016 to 2020) [18].

For tiotropium, we assumed that 28% of patients discontinued the treatment after 16 weeks [19]. Patients who discontinued (non-adherent) treatment had the exact costs and clinical outcome values as adherent patients for the first 16 weeks. Still, these input values change to those of the placebo group for all transition cycles after 16 weeks. Sensitivity analyses of the percentage of non-adherents and response rate were made, estimating each value’s upper and lower range by adding or subtracting 25% of the value defined previously.

All costs of each health state defined in the Markov model were extracted from a previously published Colombian-based study [20]. Briefly, this study identified the asthma-related direct and indirect costs of 1131 patients with severe asthma from January 1, 2004, through December 31, 2014, in Colombia. Asthma severity classification was mainly based on the paper of Jacob et al. [21]. This group of patients with severe asthma had an average of 1.4 ED visits per year and 2.5 hospitalizations per year, rates that are comparable to those reported in clinical trials and observational studies in patients with severe asthma and tiotropium use [8, 13]. Unit costs of tiotropium were taken from the National Drug Price Information System (SISMED, 2023). All costs were transformed to 2023 using official inflation data in Colombia. We use US dollars (Currency rate: US$ 1.00 = COP$ 4,000) to express all costs in the study [18].

Net Benefit: Net benefit is a calculation that puts Discounted Lifetime Costs (U$) and Discounted Lifetime QALYs onto the same scale. This is done by calculating the monetary value of Discounted Lifetime QALYs using a simple multiplication, i.e., QALYs * lambda, where: Net benefit for a strategy = QALYs * 5180 − Cost (US$). This is particularly useful when comparing several strategies because the analyst and decision maker can see the expected net value of each strategy in one single measure rather than looking at many comparisons of incremental cost-effectiveness ratios between different options. Under the rules of decision theory, the strategy with the most significant expected net benefit is optimal.

### Sensitivity analyses

To explore the parameter uncertainty of the model inputs, we conducted a probabilistic sensitivity analysis by randomly sampling from each parameter distribution (beta distribution in the case of relative risk and utilities, transition probabilities, and gamma distribution in the case of costs). The expected costs and QALYs for each treatment strategy were calculated using the model’s combination of parameter values. This process was replicated one thousand times (i.e., second-order Monte Carlo simulation) for each treatment option, resulting in the expected cost-utility. All analyses were made in Microsoft Excel®.

## Results

The mean incremental cost of tiotropium + ICS + LABA versus ICS + LABA is US$−4.65. This suggests that tiotropium is less costly. The incremental cost is uncertain because the model parameters are uncertain. The 95% credible interval for the incremental cost ranges from US$−34.44 to US$11.07. The probability that tiotropium + ICS + LABA is cost-saving compared to ICS + LABA is 0.66, Table 2.

**Table 2:** CE data/statistics

Threshold (US$ per QALY)	5180
Intervention	Tiotropium + ICS + LABA
Comparator	ICS + LABA
Number of PSA runs	10,000
Mean incremental. Effect per person (QALY)	0.0018
Mean incremental. Cost per person (US$)	−4.65
ICER estimate (US$ per QALY)	−2637.59
2.5th centile for inc. effects (QALY)	−0.048
97.5th centile for inc. effects (QALY)	0.018
2.5th centile for inc. costs (US$)	−34.44
97.5th centile for inc. costs (US$)	11.07
Probability intervention is cost saving	0.66
Probability intervention provides more benefit	0.76
Probability that intervention is cost-effective against comparator	0.77

The mean incremental benefit of tiotropium + ICS + LABA versus ICS + LABA is 0.0018 QALYs. This suggests that tiotropium + ICS + LABA is more beneficial. Again, there is uncertainty in the incremental benefit due to delay in the model parameters. The 95% credible interval for the incremental benefit ranges from − 0.048 QALYs to 0.018 QALYs. The probability that tiotropium + ICS + LABA is more beneficial than ICS + LABA is 0.76.

The expected incremental cost per QALY (ICER) is estimated at US$–2637.59. There is a probability of 0.77 that tiotropium + ICS + LABA is more cost-effective than ICS + LABA at a threshold of US$5180 per QALY.

The strategy with the highest expected net benefit is Tiotropium, with an expected net benefit of US$800.33 (equivalent to a net benefit on the effectiveness scale of 0.15 QALYs). Net benefit and 95% credible intervals for all strategies are presented in Table 3.

**Table 3:** Summary of absolute net benefit statistics

	ICS + LABA	Tiotropium + ICS + LABA
Mean discounted lifetime QALYs	0.1714	0.1732
Mean discounted lifetime costs (U$)	101	96
Expected net benefit at US$ 5180 per QALY	786	800
95% lower CI (on costs scale)	390	357
95% upper CI (on costs scale)	1070	1111
Expected net benefit on effects scale	0.1518	0.1545
95% lower CI (on effects scale)	0.0754	0.0690
95% upper CI (on effects scale)	0.2066	0.2147

### Sensitivity analyses

**Fig. 3:**
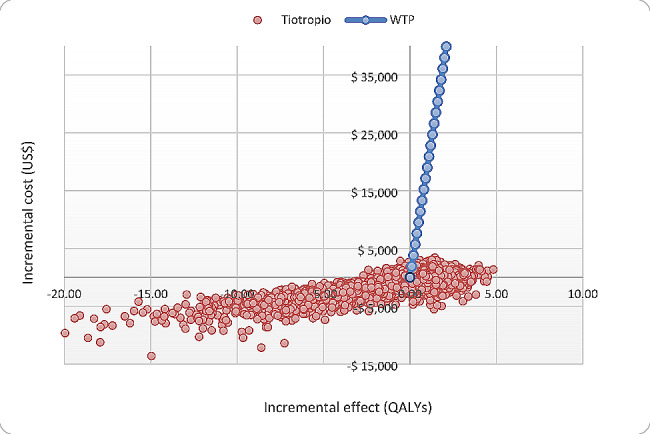
Cost-effectiveness plane

In the deterministic sensitivity analyses, our base-case results were robust to all assumptions and parameter variations. For none of the variables evaluated, variations within the established ranges led to the incremental cost-effectiveness ratio being higher than the WTP, Fig. 3.

### Cost-effectiveness acceptability curve

**Fig. 4:**
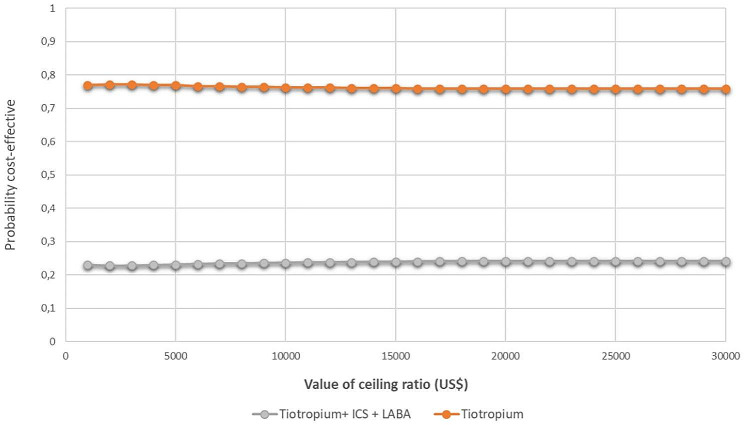
Cost-effectiveness acceptability curve

This graph shows the cost-effectiveness acceptability curve for the comparison of strategies. The results show that at a threshold value for cost-effectiveness of US$5180 per QALY, the strategy with the highest probability of being most cost-effective is Tiotropium, with a probability of 0.7683, Fig. 4.

## Discussion

This cost-effectiveness study estimates cost and QALY outcomes over a lifetime horizon for a hypothetical cohort of patients with uncontrolled asthma who had tiotropium added to their usual controller therapy. Our findings suggest that add-on therapy with tiotropium achieves better outcomes at a lower cost than standard treatment.

Our results are in line with previous studies. Hyng et al., using a similar Markov model as our study, tiotropium is a cost-effective alternative with an ICER of $4,078/QALY in frequent SABA users and $8,332/QALY on patients with poorly controlled asthma [13]. Despite our model having the same health states and using the same relative risk, the healthcare systems in Colombia and Korea are different, leading to varying medical expenses. Indeed, our costs per event of OCR bust, ED visit, or hospitalization were 69%, 79%, and 46% less, respectively, than in Korea. Another difference is the higher incidence rate of hospitalization due to exacerbation in the Hyng study concerning our study. The target population of the Hyng study was elderly patients, but in our research, it was general adults, which can explain their higher rates. Willson et al., using a six Markov model health states, estimate an incremental cost-effectiveness ratio of £21,906 per QALY gained by being tiotropium cost-effective in the UK [14]. Despite our differences in the Markov model, relative risk, utilities, and cost, in this study, the target population was a general adult, and their incidence rates were similar to our research. As is expected, the cost per event of OCS bust, ED visit, or hospitalization was higher five times in the UK than in our study in Colombia, and this can explain the differences in the magnitude of ICER between the studies. Zafari et al., also using a probabilistic Markov model with a 10-year time horizon and from a US societal perspective, found ICER of add-on therapy with tiotropium versus standard treatment, and omalizumab versus tiotropium was $34,478/QALY, and $593,643/QALY, respectively [22]. Despite methodological differences between our and this study, such as the number of health states in the model, higher cost of drugs and another direct cost in the US, and utilities, our conclusion is the same. One difference between our study and previous studies was the values of the utilities. The two previous studies use the utilities established in the Wilson study, which estimated them in the “PrimoTinAasthmatrial” population using the EuroQol EQ-5D tool in the UK population. We decided to use those reported in a systematic review to have broader values and more diverse populations. Variations in the importance of these utilities in the probabilistic sensitivity analysis did not significantly change the calculated ICER. Indeed, after 10,000 simulations in our PSA, tiotropium tends to be associated with lower costs and higher QALY; 84% of simulations were graphed in quadrants one or two of the cost-effectiveness plane.

A not minor difference in our evaluation from previous studies is that we have not only estimated the ranges of relative risks and transition probabilities using data from real-life studies but have adjusted our estimates for tiotropium adherence. Assuming 100% adherence is unrealistic and tends to overestimate the effect of tiotropium. On the other hand, including real-life study data increases the external validity of the estimates themselves compared to just basing model inputs on data from controlled clinical trials. The probabilistic sensitivity analyses’ results confirm the model results’ robustness. Since transition probabilities and utilities do not come from the Colombian population, they were subjected to probabilistic sensitivity analysis as detailed below as recommended by the Consolidated Health Economic Evaluation Reporting Standards (CHEERS) Statement [17].

The relevance of the findings regarding the cost-effectiveness of tiotropium as an add-on therapy to inhaled corticosteroids (ICS) and long-acting beta-2 agonists (LABA) for patients with severe asthma can have significant implications for healthcare decision-makers, clinicians, patients, and the broader healthcare system. Here are several vital points to consider. For clinical decision-making, our findings show that adding tiotropium to the standard ICS/LABA therapy is cost-effective and provides valuable guidance for clinicians, helping them make informed treatment choices that optimize patient outcomes. Patients with severe asthma can benefit from more effective and cost-efficient treatment with a treatment offering better symptom control, reduced exacerbations, and improved quality of life. Another point of view is that cost-effectiveness data can guide policymakers and healthcare administrators to allocate resources efficiently. Our findings may justify the inclusion of the drug in treatment guidelines and reimbursement policies. The cost of medications can be a significant barrier to access for patients. If tiotropium is shown to be cost-effective, it may make the treatment more affordable and accessible for patients with severe asthma. The findings of a cost-effectiveness study contribute to the evidence base, allowing for more informed and evidence-based clinical practice. Positive results also encourage further research into tiotropium and its role in asthma management, potentially leading to new insights and improved treatment strategies. In summary, the relevance of the findings of tiotropium’s cost-effectiveness as an add-on therapy for severe asthma lies in its potential to inform clinical practice, healthcare policies, resource allocation, and patient access to effective treatments. This kind of cost-effectiveness studies are crucial in optimizing healthcare delivery and improving patient outcomes.

Our study has some limitations. We use utilities extracted from the literature and not estimated directly from our population. As was mentioned previously, the reliability and robustness of the results were evaluated by sensitivity analyses. Our result only refers to a patient with severe asthma uncontrolled by medium-dosage to high-dosage inhaled corticosteroids plus long-acting β_2_-agonists. It cannot be extrapolated to patients with daily oral corticosteroids. Studies of tiotropium in asthma have recruited both allergic and non-allergic asthma patients. Using evidence from such trials, we assumed the same health benefits of tiotropium for allergic and non-allergic asthma patients. This assumption is supported by trials of tiotropium, which showed no difference between allergic versus non-allergic subjects [8].

In conclusion, add-on therapy with tiotropium was cost-effective when added to usual care in patients who remain uncontrolled despite treatment with medium or high-dose ICS/LABA. Our study provides evidence that decision-makers should use to improve clinical practice guidelines.

## Data Availability

(2021). DB Tiotropium [Data set]. Zenodo. 10.5281/zenodo.4763124.

## Abbreviations

ICS: inhaled corticosteroids
LABA: long-acting b2 agonist
OCS: oral corticosteroids
LAMA: long-acting muscarinic antagonists
QALYs: quality-adjusted life-years
SOC: standard therapy
WTP: willingness to pay
RR: relative risk
CHEERS: Consolidated Health Economic Evaluation Reporting Standards
SABA: Short-acting beta-agonists
ICER: Incremental cost-effectiveness rate
